# Annual Report of the Supervising Surgeon of the U. S. Marine Hospital Service

**Published:** 1874-04

**Authors:** 


*Annual Report of the Supervising Surgeon of the United States Marine Hospital Service, for the year* 1873. By John M. Woodworth, M. D.

This volume contains a statement of the work of the service for the year ending June 30th, 1873. From it we learn that 13,529 sick and disabled seamen were furnished with relief, 12,697 being maintained in the Hospital. The hospital dues collected during the year amounted to $335,845 95, which is an increase of over $12,000 over the collections of last year; due to the more faithful administration of the service. The report contains several papers of professional interest, and is also illustrated to considerable extent. A case of Double Diaphragmatic Rupture and Hernia, by Thos. C. Minor, M. D., is of considerable interest, and is well reported. The reports on Yellow Fever, are also of much value, and are carefully and thoroughly made. The Marine Hospital Service is one of much importance and under the present supervision is doing a valuable work.

